# A comparative analysis of complementary therapies use among patients attending diabetic clinics in Taiwan: 2007 vs. 2023

**DOI:** 10.1186/s12906-023-04299-9

**Published:** 2023-12-13

**Authors:** Hsiao-Yun Chang, Yu-Yao Huang, Chin-Jung Chung, Feng-Hsuan Liu

**Affiliations:** 1 Department of Nursing, Chang Gung University of Science and Technology, Taoyuan, Taiwan; 2 Division of Endocrinology and Metabolism, Department of Internal Medicine, Linkou Chang Gung Memorial Hospital, Taoyuan, Taiwan; 3 College of Medicine, Chang Gung University, Taoyuan, Taiwan

**Keywords:** Diabetes, Complementary and alternative medicine, Comparison

## Abstract

**Background:**

We conducted a comparative study to examine the differences in the use of complementary therapies (CT) among patients who attended diabetic clinics for follow-up treatment between 2007 and 2023 in Taiwan.

**Methods:**

This study employed a cross-sectional survey design to recruit individuals with diabetes from two regions (northern and southern) of Taiwan. A total of 183 and 307 participants were included in the surveys of 2007 and 2023, respectively. The data were analyzed using IBM SPSS Statistics version 28.0 to compare the survey results between the two time periods.

**Results:**

Among the various CTs, nutritional supplements remained the most prevalent, with a significant increase in usage from 68.3% in 2007 to 89.9% in 2023. Conversely, other therapies, such as Chinese herbal medicines, manipulative-based therapies, supernatural healings, and bioelectromagnetic-based therapies, demonstrated a significant decrease in usage between the two time periods. Furthermore, the disclosure rate of CT use to healthcare professionals remained persistently low, with only 24.6% in 2007 and a slight increase to 30.3% in 2023.

**Conclusion:**

The significant rise in the use of nutritional supplements in conjunction with conventional medicine, without adequate monitoring and guidance from healthcare professionals, poses a substantial risk of unregulated blood sugar control, compromised diabetes management, and potential harm to health outcomes.

## Background

The global prevalence of diabetes has increased from approximately 6.4% in 2010 to 9.3% in 2019, impacting a staggering 463 million adults worldwide. Projections suggest that this prevalence will continue to escalate, reaching 10.2% (578 million) by 2030 and 10.9% (700 million) by 2045 [1]. Middle-income countries, including Taiwan, are expected to face the highest increase in diabetes prevalence compared to high- and low-income nations, with an estimated growth rate of 21.1%. [2]. Managing diabetes and its associated health expenditures has become a major public health concern. The chronic nature of this disease, along with its potential for debilitation and long-term complications, has prompted individuals with diabetes to explore complementary therapies (CT) as a means of managing their symptoms and improving overall well-being [3, 4]. Research has shown that a significant proportion of individuals with diabetes, approximately 51% globally, engage in CT use. Among CTs, herbal and nutritional supplements are particularly common [3]. These findings highlighted the increasing recognition of CT as a potential complementary therapy in diabetes management while patients undergo conventional treatment. The field of CT is continuously evolving, and understanding the changing patterns of CT use is crucial for healthcare professionals and policymakers to tailor appropriate interventions accordingly, recognizing the importance of managing potential interactions between CT and conventional medication to optimize treatment outcomes and culturally sensitive care.

A comparative analysis of CT use trends among adults in the United States in 2002, 2007, and 2012 revealed that dietary supplements remained the most prevalent therapy, maintaining their prevalence over the years. Additionally, there was a consistent and gradual increase in the use of practices like yoga, tai chi, and qi gong across the three-time points [5]. In Korea, an 8-year follow-up study showed a slight increase in usage from 35.7% in 2006 to 38.0% in 2014 in patients with inflammatory bowel disease. Herbal therapies were the most commonly utilized CT [6]. In contrast, a study conducted in Taiwan reported a decline in CT use among adults from 48.9% in 2007 to 37.8% in 2011 and identified Chinese medicinal herbs as the most commonly utilized CT [7]. A noteworthy trend that emerged in the use of CT between 2007 and 2011 was a significant increase in the use of nutritional supplements and massage, while there was a substantial decrease in the use of Tuina (a form of Chinese therapeutic massage) and Chinese medicinal herbs [7]. These findings highlight diverse trends in CT use between the United States, Korea, and Taiwan, emphasizing the various preferences and practices related to CTs in different populations.

Research into exploring the changes and trends in CT use among individuals with diabetes over a significant period is limited. Understanding these changes can provide insights into CT use’s changing landscape, identify potential disparities, and offer a deeper understanding of the intersection between CT and diabetes management. It is of utmost importance for healthcare professionals and policymakers, particularly in the context of diabetes. By comprehending these trends, they can effectively support patients in making informed decisions about their treatment options and ensure comprehensive and patient-centered care. Therefore, the purpose of this study was to examine the differences in the patterns of CT use, their experience of CT use, and their concomitant use of CT with conventional treatments among patients who attended diabetic clinics for follow-up treatment between 2007 and 2023 in Taiwan.

## Methods

### Study design and sample

This comparative study utilized datasets from two cross-sectional surveys, both employing a structured interview approach, conducted in conventional hospitals located in the Northern and Southern regions of Taiwan during two distinct periods. The first survey was conducted from July 2006 to February 2007, and the second from October 2022 to March 2023. The participant selection criteria for the 2006 survey was originally outlined in Chang et al. [8]. To maintain uniformity in participant selection criteria across both the 2006 and 2022 surveys, we adopted the criteria established in the second survey when extracting data from Chang et al.‘s study.

A convenience sampling method was employed, and participants were included based on the following criteria: (1) individuals over the age of 20, (2) a diagnosis of diabetes, and (3) utilization of complementary and alternative medicine (CAM). To determine the sample size, the G*Power 3.1.9.7 software was used for proportions, specifically for inequality between two independent groups at the post hoc test. This analysis resulted in a total sample size of 490 participants, ensuring a high statistical power (1–β = 0.998), and the sensitivity test confirmed an acceptable effect size.

### Survey instrument

The instrument used in the second survey closely mirrors the one established in the original survey conducted by Chang et al. [8], which consisted of three main sections: demographic characteristics, patterns of CT use, and experiences of CT use. In the first section, participants provided demographic information, including age, gender, education, marriage, employment, and disease duration. The second section of the instrument gathered information on the use of 10 specific CTs. The selection of these 10 CTs was based on the findings from the initial survey conducted by Chang et al. [8] and the categories of the National Center for Complementary and Integrative Health [9], as indicates in Table 1. The third section focused on experiences of CT use and covered topics such as the reasons for starting CT use, knowledge of CT ingredients, decision makers of CT use, the administration of CT with conventional treatments, the disclosure of CT use to healthcare professionals, and the reasons for non-disclosure or reactions of disclosure from healthcare professionals.

**Table 1:** The frequency and content of CTs used by participates in 2023

Rank	Types of CT	Common Therapies^1^ (times)
1	Nutritional supplements	Glucosamine (100), multivitamin (92), vitamins D, B, C (82), Calcium (73), fish oil (57), lutein (64), probiotics (30), bitter melon (13)
2	Chinese herbal medicines	Combination herbs (36), Scientific Chinese medicine (13), ginseng (3), ganoderma (4)
3	Manipulative based therapies	Massage (11), acupucture (9), Tui-na (10), Gua-sha (6), knife therapy (2)
4	Supernatural healings	Bai-bai (15), pray (8), chanting scripture (2)
5	Exercise	All types of walking (30), fitness (10), hiking (5), jogging (3), Gi-gone (2), Tai-chi (2)
6	Diet modification	Protein-related (26), on diet (24), grains meals (4), Intermittent fasting (4),
7	Bioelectromagnetic-based therapies	Magnetic fields (3), pulsed fields (3)
8	Mind-body therapies	Relaxation (3), Meditation (2), yoga (2)
9	Non-Chinese herbs	Bilberry (5), aloe vera (2),
10	Aromatherapy	Aroma (6)

^1^ Only reported frequent use of CT among participants

### Data collection procedures

The data from the first survey conducted by Chang et al. [8] was used as secondary data in our study. We have meticulously designed the criteria for selecting and utilizing this data to ensure accuracy and reliability while minimizing potential sources of bias. Data collection methods in both surveys were replicated from the methods detailed in Chang et al.’s study [8]. Potential participants were selected by research assistants (RAs) from the appointment list. Upon confirming that the potential participants met the inclusion criteria, the RAs provided them with the information sheet and consent form, which were thoroughly discussed and signed. The RAs then conducted structured interviews, meticulously recording all the participants’ responses on the designated survey instrument.

### Statistical analyses

The collected data were reviewed for completeness, and subsequently coded and entered into the IBM SPSS Statistics version 28.0 software. To ensure data accuracy, thorough checks were conducted for outliers and any errors in coding and data entry were rectified. Descriptive statistical procedures were then employed to summarize the demographic and clinical characteristics of the study sample. Inferential statistics (Chi-square test & an independent t-test) were employed to examine the differences in sample population characteristics, patterns of CT use, and the experiences of CT use between 2007 and 2023. Since participants’ ages and the duration of diabetes differed between the two surveys and correlated to each other (*r* = 0.30), we used binary logistic regression to adjust for age to assess changes in CT use over time. The level of statistical significance for all analyses was set at a minimum of p < 0.05.

#### Ethical consideration

The Research Ethics Committees approved the first survey study by Griffith University (No.NRS/06/06/HREC) and the second survey study by Chang Gung Medical Foundation (No.202102201B0C601). Written informed consent to participate in the study was obtained before proceeding with the survey.

## Results

### Characteristics of sample

Characteristics of the sample are presented in Table 2. Most participants were female, married, retired/unemployed, older adults with at least a high school education, and diagnosed with diabetes less than ten years before the study. In a comparison of sample characteristics between both surveys, we observed participants’ age and duration of diabetes in 2023 who were older and had a longer duration of diabetes. Other characteristics of gender, education, marriage, and employment were not different between both sample populations.

**Table 2:** Demographic Characteristics of the Overall Sample

Characteristics	2007 (*N* = 183)		2023 (*N* = 307)			
	M	SD	M	SD	t	*p*
Age	58.54	12.05	64.6	10.99	-5.69	< 0.01
Duration of diabetes	8.28	7.06	11.21	8.95	-3.78	< 0.01
	*N*	%	*N*	%	χ^2^	
Gender					0.28	0.59
Male	76	41.5%	120	39.1%		
Female	107	58.5%	187	60.9%		
Highest Education					1.87	0.17
< High school	80	43.7%	115	37.5%		
≥ High school	103	56.3	192	62.5%		
Marriage					1.00	0.32
Single	13	7.1%	30	9.7%		
Being Married	170	92.9%	278	90.3%		
Employment					1.76	0.18
Employed	56	30.6%	112	36.5%		
Unemployed	127	69.4%	195	63.5%		

### Changes in the pattern of CT use between 2007 and 2023

Table 3 presents the frequency of CTs used by participants in the 2007 and 2023 surveys. Throughout both periods, nutritional supplements emerged as the most commonly used therapy, exhibiting a notable relative increase from 68 to 89.9%. Conversely, several CTs showed a significant decrease in usage proportions in the 2023 survey compared to 2007. These included Chinese herbal medicines (36.1% vs. 14.3%), manipulative-based therapies (20.8% vs. 7.2%), supernatural healings (17.5% vs. 8.5%), and bioelectromagnetic-based therapies (10.9% vs. 1.7%). In contrast, the proportions of individuals utilizing exercise, diet modification, cupping and scraping, mind-body therapies, and non-Chinese herbs did not show significant differences between both surveys. Notably, aromatherapy increased from 0% in 2007 to 2.0% in 2023. The use of multiple combinations of CT (over two types of CT) was reported by 53.6% in 2007 and significantly decreased to 32.2% in 2023 (χ^2^ = 21.65, *p* < 0.001). In addition, Table 1 illustrates the common therapies used under each type of CT.

**Table 3:** Comparative 12-month frequency of CT use between 2007 (*N* = 183) and 2023 (*N* = 307)

Therapies	2007 Users	2023Users				
	*N*%	*N*%	Wald	Exp(B)	*p*	95% CI
Nutritional supplements	125(68.3)	275(89.9)	24.63	3.52	< 0.001	2.14 ~ 5.78
Chinese herbal medicines	66(36.1)	44(14.3)	27.34	0.30	< 0.001	0.019 ~ 0.47
Manipulative based therapies	38(20.8)	22(7.2)	13.34	0.34	< 0.001	0.19 ~ 0.61
Supernatural healings	32(17.5)	26(8.5)	6.49	0.47	0.011	0.27 ~ 0.84
Exercise	26(14.2)	51(16.7)	1.99	1.47	0.158	0.86 ~ 2.52
Diet modification	25(13.2)	46(15.0)	0.64	1.25	0.426	0.72 ~ 2.16
Bioelectromagnetic-based therapies	20(10.9)	5(1.7)	15.05	0.13	< 0.001	0.05 ~ 0.37
Mind-body therapies	9(4.9)	7(2.3)	1.82	0.49	0.177	0.18 ~ 1.38
Non-Chinese herbs	7(3.8)	4(1.4)	1.48	0.46	0.227	0.13 ~ 1.63
Aromatherapy	0(0)*	6(2.0)	-	-	-	-

* Cells have an expected count of less than 5

### Patients’ experiences of CT use

In 2007, the primary reason for initiating CT use among participants was a personal belief in CT (43.5%), while in 2023, the influence of people around them who believed in CT (40.7%) emerged as the main factor (refer to Table 4). Additionally, there was a significant increase in the proportion of individuals starting CT use based on healthcare professionals’ recommendations (6.5% vs. 15.6%), whereas there was a significant decrease in individuals opting for CT due to feeling safer and experiencing fewer side effects (8.8% vs. 2.9%). In the 2007 and 2023 surveys, the primary source of CT information remained families, with proportions of 49.2% and 42.9%, respectively (see Table 4). Notably, there was a substantial increase in the utilization of media as a source of CT information, with percentages rising from 11.3% in 2007 to 23.7% in 2023, whereas there was a significant decrease in the reliance on friends as a source of CT information, with proportions declining from 28.3 to 19.3%, respectively. The primary decision on CT use was predominantly made by the participants themselves, with proportions of 75.4% in 2007 and 85.0% in 2023. There was a significant decrease in the proportion of decisions made by family members, declining from 21.3% in 2007 to 13.7% in 2023.

**Table 4:** Comparison of experience on CT use between 2007 and 2023

	2007 Users		2023 Users			
	*N* = 183	%	*N* = 307	%	χ^2^	*P*
The reasons for initial CT use					15.74	0.03
Others believe in CT	74	40.3	125	40.7		
I believe in CT	80	43.5	122	39.7	®	
Recommend by HCP^1^	12	6.5	48	15.6	〈	
Feel safer, less side-effect	16	8.8	9	2.9	®	
Dissatisfaction with medicine	2	0.9	3	1.0		
Sources of CT information	*n* = 203		*n* = 393		20.41	< 0.001
Family	90	42.5	135	34.3	®	
Friend	60	28.3	76	19.3	®	
Professionals	31	14.6	66	16.8	〈	
Medical journal	7	3.3	23	5.9	〈	
Media	24	11.3	93	23.7	〈	
Decision makers of CT use					7.53	0.023
Own decision	138	75.4	261	85.0	〈	
Family & friend’s decision	39	21.3	42	13.7	®	
Professionals	6	3.3	4	1.3	®	
Knowledge of CT ingredients					2.51	0.113
Known	60	32.8	122	39.7		
Unknown	123	67.2	185	60.4		
How to mixed					8.28	0.016
No change	53	29.0	113	36.8	〈	
Separately taken	107	58.5	190	61.9	®	
Reduce or stop the medicine	23	12.5	4	1.3	®	
Disclosure of CT					1.84	0.175
No	138	75.4	214	69.7		
Yes	45	24.6	93	30.3		
Reactions of disclosure from HCP	*N*=	45	*N*=	93	8.77	0.012
Encourage you	15	35.7	38	40.9	〈	
Discourage	9	16.7	4	4.3	®	
Up to you	21	47.6	51	54.8	〈	
Reasons for non-disclosure^2^	*n* = 241		*n* = 254		70.04	< 0.001
Never thought	77	32.0	58	22.8	®	
Felt safe	68	28.2	49	19.3	®	
No asked by HCP	30	12.4	118	46.5	〈	
No time to discuss	5	2.1	27	10.6	〈	
Afraid of HCP and no acquaintance	16	6.6	2	0.8	®	

^1^HCP: Healthcare professionals; ^2^The question allowed multiple responses

### CT knowledge among users

The majority of CT users in both the 2007 and 2023 surveys lacked knowledge about the ingredients of the CT products they were using, with proportions of 67.2% and 60.4%, respectively (see Table 4). Consequently, most participants opted to take their conventional medication and CT at separate times to minimize potential interactions (58.5% vs. 61.9%). Notably, there was a significant increase in the proportion of participants choosing to maintain the same timing for administering their conventional medication (29.0% vs. 36.8%), while a significant decrease was observed in the proportion choosing to either reduce or discontinue the dose of their conventional medications (12.5% vs. 1.3%).

### Decisions regarding disclosure of CT use

Only 24.6% in 2007 and 30.3% in 2023 of participants who sought guidance from healthcare professionals regarding their CT use in conjunction with conventional medicines, representing a significant increase (see Table 4). Among those who disclosed their CT use, there were differences in the responses received from healthcare professionals between the two periods. Firstly, a substantial proportion of healthcare professionals in both 2007 and 2023 (47.6% and 54.8%, respectively) stated that the decision to use CT was solely the patient’s responsibility and offered no specific comments regarding CT use. Secondly, a considerable percentage of healthcare professionals in both periods (35.7% in 2007 and 40.9% in 2023) encouraged the patients to incorporate CT into their treatment. However, there was a significant change in the proportion of healthcare professionals who warned patients about the potential side effects of CT and discouraged its use, with a decline from 19.7% in 2007 to 4.3% in 2023.

Changes in the reasons provided by participants for not disclosing their CT use to healthcare professionals were observed. These changes included a decrease in the proportion of participants who never thought of informing their healthcare professionals (32.0% in 2007 to 22.8% in 2023), who believed CT use is safe and therefore saw no need to discuss it (28.2% in 2007 to 19.3% in 2023), and who thought healthcare professionals would discourage CT use due to inadequate knowledge of CT (6.6% in 2007 to 0.8% in 2023). A significant increase in the proportion of participants reported that healthcare professionals did not inquire about their CT use (12.4% in 2007 to 46.5% in 2023), and they felt insufficient time to discuss CT use (2.1% in 2007 to 10.6% in 2023).

## Discussion

This study provides a comprehensive analysis of the patterns, experiences, and communication of CT use among individuals with diabetes over time, thereby enhancing our understanding of evolving trends in CT use and its potential clinical implications. Nutritional supplements emerged as the most frequently used therapy throughout both time periods, indicating their widespread popularity among individuals with diabetes. The substantial increase in their usage, reaching 89.9% in 2023, signifies a growing reliance on nutritional supplements as a complementary therapeutic approach for diabetes management. This finding aligns with a previous comparative study among adults in Taiwan [7]. However, our study revealed notable declines in the usage of Chinese herbal medicines, manipulative-based therapies, supernatural healings, and bioelectromagnetic-based therapies. These findings reflect shifts in patient preferences and evolving the landscape of CT use among individuals with diabetes in 2023, compared to the trends observed in the earlier Taiwan study from 2007 to 2011 [7]. Additionally, the stability of exercise, diet modification, cupping and scraping, mind-body therapies, and non-Chinese herbs suggests their continued relevance in managing diabetes. The change in trend observed in 2023 regarding CT used by individuals with diabetes can be influenced by several factors: a substantial increase in the availability of health information [10], shifts in societal attitudes toward health and wellness after COVID-19 [11], the availability of new research and clinical evidence, and recommendations of healthcare providers [12].

A significant shift in the experiences of CT use among individuals with diabetes was observed between 2007 and 2023. The primary reasons for initiating CT use shifted over time, with a significant increase in the proportion of participants who started using CT based on healthcare professionals’ recommendations. This suggests a growing acceptance and recognition of CT by healthcare professionals, who may incorporate these therapies into their treatment recommendations [13]. The primary sources of CT information shifted towards media platforms while decreasing reliance on friends as influential sources. Particularly during the COVID-19 pandemic, the accessibility of information through digital media has played a significant role in shaping people’s choices [14]. This finding was also observed in the increasing autonomy of individuals in making decisions about CT use and reduced reliance on family members for guidance in this regard [15].

A significant concern raised by our study is the limited knowledge of individuals with diabetes regarding the ingredients of CT products they are using across these years. This highlighted a potential gap in understanding the components and potential interactions of these therapies among them. Consequently, participants tend to prioritize preventing potential interactions between their conventional medication and CT by adjusting their medication schedules rather than altering or stopping the dosage of their conventional medication. Although the disclosure of CT use to healthcare professionals remained low, our study findings revealed an increase in participants expressing reasons for not disclosing their CT use, particularly citing limited time for discussion and healthcare professionals’ lack of inquiry regarding CT use. This indicates a potential barrier to effective communication between patients and healthcare professionals regarding CT use [16]. Those who disclosed CT use received varying responses from healthcare professionals. While most professionals offered no comment on CT use or regarded it as solely the patient’s decision, there was also an increase in healthcare professionals who encouraged CT use. This may be attributed to the growing awareness and acceptance of CT within the healthcare system [17].

Overall, a shift towards specific therapies, such as nutritional supplements, suggests the importance of regularly monitoring and understanding CT trends to inform healthcare professionals and policymakers in delivering comprehensive and patient-centered care regarding the evidence and safety profiles of various CT therapies to provide accurate information, guidance, and support to individuals with diabetes in their decision-making process regarding CT use. Additionally, a growing trend toward individuals taking a more proactive role in their healthcare choices highlights the importance of empowering patients in decision-making processes related to CT use. It underscores the need for healthcare professionals to engage in open and non-judgmental discussions with patients about their CT use, taking into account their beliefs, preferences, and the influence of social circles. Furthermore, there is a need for healthcare professionals to actively inquire about CT use, educate patients about the potential interactions and ingredients of CT products, and guide the appropriate timing and dosage of both conventional medicine and CT. Enhancing patient education and awareness about CT can help individuals make informed decisions and facilitate the safe and effective integration of CT into their conventional treatment regimens. Finally, healthcare professionals should remain vigilant in monitoring patients’ CT use and be prepared to address any concerns or questions that arise to ensure comprehensive and patient-centered care.

### Limitations

While the study provides valuable insights into the use of CT among individuals with diabetes, some potential biases and limitations should be acknowledged, such as selection bias, social desirability bias, and limited assessment of CT. The selection bias was caused by participants who were already predisposed to using CT, and their experiences may not represent the general population. However, our findings regarding the changes in the pattern of CT use were consistent with the previous Taiwanese study [7]. The data on CT use was collected through self-report, which may introduce social desirability bias. This is because participants may be reluctant to report CT use that healthcare professionals believe is unacceptable. During data collection, ensuring participants that their responses were kept confidential and that there were no right or wrong answers could encourage more honest reporting of CT use. As socioeconomic and severity of diabetes factors can play a crucial role in healthcare decision-making, future studies should consider incorporating measures of economic status to provide a more comprehensive understanding of the factors influencing CT choices among patients with diabetes.

## Conclusion

The study revealed changes in the pattern of CT use among individuals with diabetes between 2007 and 2023. Nutritional supplements emerged as the most commonly used therapy across these years, while others experienced varying levels of decline or stability. The reasons for initiating CT use and the sources of information shifted over time, suggesting evolving attitudes and influences. These findings emphasize the need for healthcare professionals to stay updated on trends in CT use, provide accurate information, and support patients in making informed decisions about CT use. Understanding the changing landscape of CT use is crucial for healthcare professionals and policymakers to ensure comprehensive and patient-centered care for individuals with diabetes. Further research is needed to explore the efficacy, safety, and potential interactions of different CTs with conventional diabetes treatments.

## Data Availability

The datasets used and/or analysed during the current study are available from the corresponding author upon reasonable request.

## Abbreviations

CT: Complementary Therapies
COVID-19: Coronavirus Disease
